# Small- to medium-sized arteritis diagnosed by F-18 FDG PET/CT: A case report

**DOI:** 10.1097/MD.0000000000048083

**Published:** 2026-03-20

**Authors:** Sang-Min Oh, Seok Tae Lim, Hwan-Jeong Jeong, Yeon-Hee Han

**Affiliations:** a Department of Internal Medicine, Jeonbuk National University Medical School and Hospital, Jeonju, Jeonbuk, Republic of Korea; b Department of Nuclear Medicine, Research Institute of Clinical Medicine of Jeonbuk National University-Biomedical Research Institute of Jeonbuk National University Hospital, Cyclotron Research Center, Molecular Imaging and Therapeutic Medicine Research Center, Jeonbuk National University Medical School and Hospital, Jeonju, Jeonbuk, Republic of Korea.

**Keywords:** arteritis, F-18 FDG, fever, PET/CT

## Abstract

**Rationale:**

Early diagnosis of arteritis is challenging due to nonspecific symptoms and often requires invasive procedures. This case illustrates the utility of F-18 FDG PET/CT as a noninvasive imaging modality for identifying inflammatory vascular diseases, thereby facilitating timely treatment and avoiding unnecessary interventions.

**Patient concerns:**

A 65-year-old man presented to the emergency department with a persistent fever and generalized muscle pain lasting for 3 weeks.

**Diagnoses:**

F-18 FDG PET/CT revealed linear and branching tree-like patterns of hypermetabolism in the arteries of both arms and thighs, leading to the diagnosis of arteritis.

**Interventions:**

Following the rheumatologist’s recommendation, intravenous prednisolone therapy was initiated.

**Outcomes:**

Shortly after starting prednisolone therapy, his fever subsided and muscle pain resolved.

**Lessons:**

This case highlights the valuable role of F-18 FDG PET/CT in diagnosing small- to medium-sized arteritis, providing a noninvasive approach that can help avoid unnecessary procedures and inappropriate use of antibiotics, while also enabling objective monitoring of treatment response.

## 1. Introduction

Fever of unknown origin (FUO) is a clinical condition defined by a fever lasting more than 3 weeks without an identifiable origin, despite appropriate evaluation during at least 3 days of hospitalization.^[1]^ It involves a wide range of potential etiologies, including infections, malignancies, autoimmune and noninfectious inflammatory diseases, and miscellaneous causes.^[2]^ The diagnostic approach to FUO remains complex, particularly in elderly patients, where underlying conditions may be multifactorial.

Traditionally, FUO is evaluated using a combination of physical examination, laboratory test, serologic markers, and conventional imaging such as chest X-ray, ultrasound, and computed tomography (CT). However, these methods often fall short in localizing the source of fever, especially when the inflammatory or infectious process is subtle, multifocal, or deeply located. Therefore, in such cases, a highly sensitive imaging modality is required to detect the source of fever that may not be identified by conventional methods.

Fluorine-18 fluorodeoxyglucose positron emission tomography/CT (F-18 FDG PET/CT) has emerged as a powerful tool in the evaluation of FUO, utilizing the high FDG uptake of inflammatory cells,^[3]^ which had previously been considered a source of false positives in the oncologic field.^[4]^ By detecting increased glucose metabolism in inflamed tissues, F-18 FDG PET/CT provides functional and anatomical information that can localize hidden disease processes. Numerous studies have demonstrated its usefulness in identifying fever foci when conventional diagnostics are inconclusive, leading to more accurate and timely treatment decisions.^[5]^

Among the various causes of FUO, vasculitis, particularly small- to medium-sized arteritis, is often underdiagnosed due to its diverse clinical presentation and lack of specific biomarkers. Although biopsy remains the gold standard for diagnosis, it is not always feasible due to the risks of invasive procedures and the variable diagnostic yield depending on the patient’s condition and the target organ.

In this report, we present a case of a 65-year-old man with FUO whose diagnosis of small- to medium-sized arteritis was established using F-18 FDG PET/CT. This case highlights the diagnostic value of F-18 FDG PET/CT in detecting vascular inflammation in the absence of definitive histopathological confirmation and demonstrates its potential as a noninvasive tool in the evaluation and management of FUO.

## 2. Case presentation

A 65-year-old man presented to the emergency department with a high fever, reaching up to 40°C and generalized muscle pain. His symptoms had begun 3 weeks earlier and worsened 3 days prior to presentation. He reported traveling to Thailand 1 month ago and participating in scuba diving. Throughout the day, he experienced multiple episodes of fever, which temporarily subsided after taking antipyretics. Aside from medication for diabetes, he reported no other medications or significant medical history. His white blood cell and platelet counts were elevated at 14,720/μL (normal range: 4800–10,800) and 547,000/μL (normal range: 130,000–450,000), respectively, while his hemoglobin level was decreased to 9.8 g/dL (normal range: 13.0–16.5). The erythrocyte sedimentation rate and C-reactive protein were markedly elevated, at 120 mm/hr (normal range <9) and 162.72 mg/L (normal range <5), respectively. Subsequent chest and abdominal CT scans revealed no abnormal findings. Furthermore, blood and urine cultures were negative for bacterial growth. All tests for malaria, influenza virus, respiratory syncytial virus, and antistreptolysin O were negative. The rapid plasma reagin test for syphilis was also nonreactive.

**Figure 1.**
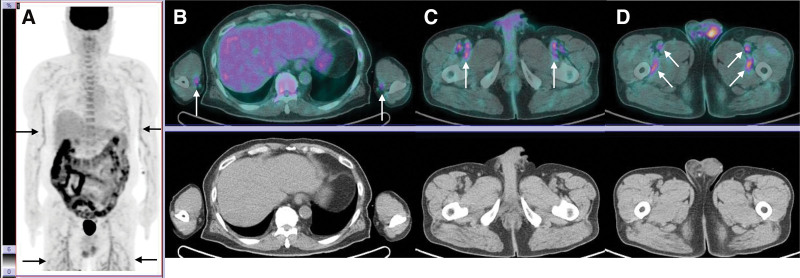
Initial F-18 FDG PET/CT. On the MIP image (A), long and linear hypermetabolism was noted in both arms (arrows), along with branching tree-like patterns of FDG uptake in both thighs (arrows). Multiple tortuous uptakes in the abdomen were physiological uptakes in the bowels. Fusion F-18 FDG PET/CT and CT images (B–D) showed high metabolism in the bilateral brachial and radial arteries, as well as bilateral external iliac, femoral, and perforating arteries (white arrows). These findings are highly suggestive of small- to medium-sized arteritis. CT = computed tomography, FDG = fluorodeoxyglucose, MIP = maximum intensity projection, PET = positron emission tomography.

To identify the cause of the fever, F-18 FDG PET/CT was performed (Fig. 1). The patient fasted for 6 hours before intravenous injection of F-18 FDG and blood glucose levels were found to be below 126 mg/dl. 5.5 MBq of F-18 FDG per kilogram of body weight was administered intravenously. Scanning was performed about 60 minutes after FDG administration. Images were obtained from the base of the skull to the proximal thigh using a Biograph TruePoint 40 PET/CT scanner (Siemens Medical Solutions, Knoxville). A CT scan was obtained first using a continuous spiral technique (120 kVp, 160 mA, 0.5 seconds rotation time). A PET scan was then acquired in a 3-dimensional mode for 2.5 minutes in each bed position. The PET data obtained were reconstructed iteratively using an ordered-subset expectation maximization algorithm (128 × 128 matrix, 3.27 mm slice thickness, subset: 21, iterations: 2).

On the maximum intensity projection image, long and linear areas of hypermetabolism were observed in both arms, accompanied by branching, tree-like patterns of FDG uptake in both thighs. Fusion F-18 FDG PET/CT images revealed increased metabolic activity in the bilateral brachial and radial arteries, as well as in the external iliac, femoral, and perforating arteries bilaterally. These findings are highly suggestive of small- to medium-sized arteritis. No abnormal hypermetabolism was seen in the temporal arteries, aorta, or aortic arch, which are sites commonly affected in giant cell arteritis. Further evaluation for the underlying cause of arteritis showed that the rheumatoid factor was within normal limits, and both antinuclear antibody (ANA) and antineutrophil cytoplasmic antibody (ANCA) tests were negative.

**Figure 2.**
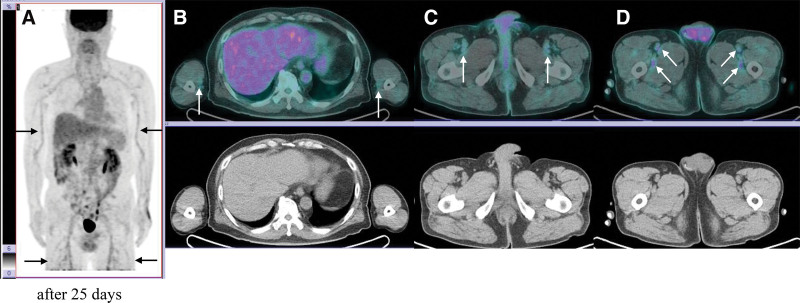
The second F-18 FDG PET/CT after prednisolone treatment. Follow-up F-18 FDG PET/CT performed 25 d after the initial scan showed significantly decreased metabolic activity in the previously affected arteries (A–D), indicating a favorable treatment response. Arrows indicate the involved arteries. CT = computed tomography, FDG = fluorodeoxyglucose, PET = positron emission tomography.

Following the rheumatologist’s recommendation, prednisolone therapy was initiated, leading to a rapid improvement in his symptoms. On follow-up F-18 FDG PET/CT (Fig. 2) performed 25 days after the initial scan, metabolic activity had significantly decreased in the previously affected arteries. The SUVmax values of the right and left arm arteries decreased from 2.88 to 1.83 and from 3.24 to 2.50, respectively. Both the initial and follow-up F-18 FDG PET/CT scans were acquired on the same scanner using an identical protocol. Regarding the patient’s prednisolone therapy, the patient received prednisolone 5 mg, 1 tablet twice daily for 25 days, followed by a tapering regimen of 5 mg once in the morning and 0.5 tablet in the evening for 23 days. The dose was then tapered to 0.5 tablet twice daily for 35 days, and subsequently to 0.5 tablet every other morning for 82 days.

## 3. Discussion

Small-to medium-sized arteritis refers to vasculitis that predominantly affects the major visceral arteries, small intraparenchymal arteries, arterioles, and capillaries.^[6]^ Its causes include viruses, bacteria, drugs, and autoimmune conditions such as polyarteritis nodosa and ANCA-associated vasculitis.^[7–9]^ Although arterial biopsy remains the gold standard for diagnosing arteritis and excluding its mimics, it is not always feasible, particularly when the involved vessels are deep-seated, not clearly localized, or when there is a high risk of uncontrolled bleeding.^[10,11]^ In such situations, F-18 FDG PET/CT can serve as a valuable noninvasive alternative, offering functional information about disease activity and extent. The findings in this case suggest that F-18 FDG PET/CT may complement or, in selected cases, reduce the need for biopsy, especially when clinical presentation and serologic markers are inconclusive.

While numerous studies have reported the clinical utility of F-18 FDG PET/CT in patients with FUO,^[12–15]^ relatively few have focused specifically on its application in arteritis.^[16]^ Although identifying the precise cause of arteritis in this patient was challenging, this case clearly demonstrates the diagnostic value of F-18 FDG PET/CT in detecting small- to medium-sized arteritis. It enabled identification of metabolically active vascular segments that would have been difficult to detect using conventional imaging modalities such as ultrasound or CT. In the present case, despite the use of both chest and abdominal CT scans, arteritis was not identified. The F-18 FDG PET/CT approach proved particularly useful, as the affected arteries did not exhibit clear structural abnormalities on CT. Furthermore, its whole-body imaging capability allowed for a comprehensive assessment of vascular inflammation without the need for invasive procedures.

Early identification of arteritis using F-18 FDG PET/CT can play a crucial role in the management of patients with FUO. In clinical practice, patients with FUO are often empirically treated with broad-spectrum antibiotics in the absence of a confirmed diagnosis, which increases the risk of inappropriate antimicrobial use, adverse drug effects, and the development of antibiotic resistance.^[17]^ By providing early and accurate detection of inflammatory conditions such as arteritis, F-18 FDG PET/CT facilitates the prompt initiation of appropriate immunosuppressive therapy, thereby avoiding unnecessary antibiotic administration. This case demonstrates the value of F-18 FDG PET/CT not only in guiding diagnosis but also in supporting more rational and effective clinical decision-making in FUO management.

Beyond diagnosis, F-18 FDG PET/CT also proved valuable in monitoring treatment response. The follow-up scan performed 25 days after initiating prednisolone therapy revealed a marked reduction in FDG uptake in the affected arteries, which correlated with clinical improvement. This supports the role of F-18 FDG PET/CT as an objective tool for evaluating therapeutic efficacy in arteritis over time. Unlike previous case reports that presented only the initial F-18 FDG PET/CT without providing quantitative parameters such as SUVmax, our report offers valuable information by demonstrating changes in arterial FDG uptake with objective parameter through F-18 FDG PET/CT performed before and after prednisolone therapy.

Although F-18 FDG PET/CT offers several advantages, as mentioned above, special caution is warranted when interpreting its findings, as several medical conditions can mimic the imaging appearance of arteritis. Increased FDG uptake along vascular structures is not specific to arteritis and may also be observed in a variety of non-arteritic conditions, including atherosclerosis,^[18]^ postsurgical or post-procedural changes,^[19,20]^ and even physiologic muscular uptake adjacent to arteries.^[21]^ These mimics can potentially lead to false-positive interpretations, particularly when clinical correlation or serologic support is lacking. Therefore, accurate diagnosis requires integration of F-18 FDG PET/CT findings with the patient’s clinical presentation, laboratory data, and, when necessary, correlation with other imaging modalities to avoid misdiagnosis and inappropriate treatment.

On the initial F-18 FDG PET/CT image in this case, extensive bowel uptake of FDG was noted, likely attributable to the patient’s metformin use. The patient was on metformin therapy during both the initial and follow-up F-18 FDG PET/CT scans; however, bowel uptake was not observed in the follow-up study. At the time of the initial F-18 FDG PET/CT, the patient was taking Metformin XR (metformin hydrochloride 750 mg) twice daily, in the morning and evening, whereas at the time of the follow-up F-18 FDG PET/CT, the patient was taking Diabex (metformin hydrochloride 1000 mg) once daily. It was presumed that the difference in the drug’s half-life might have influenced bowel uptake of FDG.

There are several limitations to this case report. First, the exact cause of the patient’s arteritis could not be identified, and the diagnosis was not histopathologically confirmed. However, the necessity of biopsy in determining the precise cause of arteritis remains controversial,^[22]^ and some studies have reported biopsy-negative cases despite strong clinical suspicion of arteritis.^[23,24]^ Second, this is a single case report, and the findings may not be generalizable to all patients with arteritis or similar clinical presentations. It should also be considered that clinical manifestations and metabolic patterns may vary among individuals. Lastly, although follow-up F-18 FDG PET/CT showed minimal to mild residual metabolic activity, a scan demonstrating complete normalization was not obtained. Nevertheless, considering that the patient’s symptoms had fully resolved, an additional F-18 FDG PET/CT was deemed clinically unnecessary due to concerns about radiation exposure.

In conclusion, this case highlights the valuable role of F-18 FDG PET/CT in diagnosing small- to medium-sized arteritis in a patient with FUO, offering a noninvasive approach that can help avoid unnecessary procedures and inappropriate use of antibiotics, while also enabling objective monitoring of treatment response.

## Author contributions

**Conceptualization:** Sang-Min Oh, Hwan-Jeong Jeong, Yeon-Hee Han.

**Data curation:** Sang-Min Oh, Seok Tae Lim.

**Methodology:** Hwan-Jeong Jeong, Yeon-Hee Han.

**Validation:** Seok Tae Lim, Hwan-Jeong Jeong.

**Writing – original draft:** Sang-Min Oh, Yeon-Hee Han.

**Writing – review & editing:** Seok Tae Lim, Hwan-Jeong Jeong.
